# Another Perspective on Huntington’s Disease: Disease Burden in Family Members and Pre-Manifest HD When Compared to Genotype-Negative Participants from ENROLL-HD

**DOI:** 10.3390/brainsci11121621

**Published:** 2021-12-08

**Authors:** Jannis Achenbach, Carsten Saft

**Affiliations:** Huntington Center North Rhine-Westphalia, Department of Neurology, St. Josef-Hospital Bochum, Ruhr-University Bochum, Gudrunstraße 56, 44791 Bochum, Germany; carsten.saft@rub.de

**Keywords:** Huntington’s disease, caregiver burden, premanifest Huntington’s disease, prodromal Huntington’s disease, ENROLL-HD

## Abstract

Background: In addition to the effects on patients suffering from motor-manifest Huntington’s disease (HD), this fatal disease is devasting to people who are at risk, premanifest mutation-carriers, and especially to whole families. There is a huge burden on people in the environment of affected HD patients, and a need for further research to identify at-risk caregivers. The aim of our research was to investigate a large cohort of family members, in comparison with genotype negative and premanifest HD in order to evaluate particular cohorts more closely. Methods: We used the ENROLL-HD global registry study to compare motoric, cognitive, functional, and psychiatric manifestation in family members, premanifest HD, and genotype negative participant as controls. Cross-sectional data were analyzed using ANCOVA-analyses in IBM SPSS Statistics V.28. Results: Of *N* = 21,116 participants from the global registry study, *n* = 5174 participants had a premanifest motor-phenotype, *n* = 2358 were identified as family controls, and *n* = 2640 with a negative HD genotype. Analysis of variance revealed more motoric, cognitive, and psychiatric impairments in premanifest HD (all *p* < 0.001). Self-reported psychiatric assessments revealed a significantly higher score for depression in family controls (*p* < 0.001) when compared to genotype negative (*p* < 0.001) and premanifest HD patients (*p* < 0.05). Family controls had significantly less cognitive capacities within the cognitive test battery when compared to genotype negative participants. Conclusions: Within the largest cohort of HD patients and families, several impairments of motoric, functional, cognitive, and psychiatric components can be confirmed in a large cohort of premanifest HD, potentially due to prodromal HD pathology. HD family controls suffered from higher self-reported depression and less cognitive capacities, which were potentially due to loaded or stressful situations. This research aims to sensitize investigators to be aware of caregiver burdens caused by HD and encourage support with socio-medical care and targeted psychological interventions. In particular, further surveys and variables are necessary in order to implement them within the database so as to identify at-risk caregivers.

## 1. Introduction

The autosomal-dominant Huntington’s disease (HD) is accompanied by progressive neurodegeneration and several different motoric, cognitive, and psychological–behavioral symptoms [1]. Although primary motor symptoms of the monogenic disease with a typical mid-life onset are associated with chorea, manifold other symptoms, such as dystonia, hypokinetic–rigidity or myoclonic symptoms, are well described [2,3,4]. Behavioral–psychiatric disturbances with depression, aggressive behavior, anxiety, apathy, or cognitive changes that decline over time can already occur in the prodromal disease phase [5,6].

Manifold approaches have been created to assess the role of caregivers and families, to find appropriate measurement and assessment methods, to evaluate quality of life and identify determinants to assess the influence of HD on patients and caregiver burdens [7,8,9,10,11]. Because findings are mostly based on case reports with qualitative interviews, further understanding and validation in a large real-world cohort is necessary.

Neuropsychiatric symptoms have been identified to decrease the quality of life in affected HD patients and increase burden in caregivers [10,12,13]. Although previous research describes the huge strain and burden on people in the environment of HD patients, other research highlights the vast necessity to identify at-risk caregivers and implement early interventions, such as targeted psychological interventions [13,14]. In this context, Yu et al. described that being a sole caregiver is associated with higher burden and caregivers are at higher risk for psychosomatic symptoms and burnout [13]. 

It is known that HD has major impacts on family systems by changing roles and there is urgent need to provide the best individual support, depending on the stage of disease and specific situation [9]. Wibawa et al. identified that psychiatric–cognitive symptoms, such as anosognosia or being unawareness of own deficits, are associated with greater caregiver burden in a cohort of *n* = 38 HD patients [15]. These psychiatric symptoms have been well described and develop frequently in HD, increasing with disease progression but being present even in prodrome HD [6]. The evaluation of neuropsychiatric symptoms in HD investigated apathy, irritability, and depression within the Problem Behaviors Assessment (PBA) scale with a notable higher longitudinal prevalence within the course of the disease [16]. Apathy, perseveration, and obsession was identified to correlate with higher motor impairments and cognitive declines where irritability and depression did not increase with disease progression [17,18]. Recently, investigations described irritability in HD as a multidimensional construct associated with other psychiatric behavioral symptoms, such as anxiety and depression [19]. Additionally, an impaired ability to reflect on the mental states of the self and others (theory of mind; ToM), impaired recognition of emotions and social cognitive deficits might impair communication and living with those individuals affected by HD [20,21,22,23,24].

Another perspective was taken and objectives pursued to analyze—in addition to early manifest HD patients—prodromal and premanifest mutation carriers, whereby neuropsychiatric symptoms were highly prevalent in premanifest stages, such as irritability, apathy, and executive dysfunctions (measured within the PBA) [25,26]. Within a small cohort of *n =* 31 participants, an association between hopelessness, depression, and anxiety symptoms was recently detected in the Hospital Anxiety and Depression Scale (HADS) in *HTT*-mutation carriers without clinical HD diagnosis when compared to participants without mutation, emphasizing the need for timely diagnostics and symptomatic treatments [27]. It remains unknown whether these findings can be validated in larger HD cohorts.

Because the evaluation of psychopathology in premanifest and affected HD patients can be very challenging, the role of caregivers is also important for providing information and assessments about psychopathologic symptoms in affected patients [28]. Therefore, dimensional tests, such as the aforementioned PBA scale, the Unified Huntington’s Disease Rating Scale (UHDRS) for functionality or cognition, and the HADS, have been identified to be reliable and sensitive instruments for documenting and monitoring symptoms [28,29,30]. Specific items of the HADS were identified to be appropriate for measuring general psychopathologic distress in HD-affected patients [30] and the PBA-short form (PBA-s) was assessed in large HD cohorts as being a reliable and valid clinical outcome measure [31,32].

The evaluation of a large caregiver cohort with regard to psychiatric, cognitive, and functional capacities for the first time helps to better understand how to provide the best individual support in terms of socio-medical care and adapting targeted psychological interventions. This research focuses on clarifying and sensitizing the large impact of HD on people, not affected themselves, but rather who are in the environment. 

We took advantage of a huge global database of HD patients, to investigate participants, such as family controls, premanifest HD, and genotype-negative participants as a control group. The aim of the study was to investigate the three groups in order to evaluate and confirm findings of neuropsychiatric abnormalities and to expand knowledge with regard to cognitive and functional capacities. Since our focus in this research is on caregivers, we did not further analyze the manifest HD group included in ENROLL-HD.

## 2. Methods 

### 2.1. Participants from the Global Registry Study ENROLL-HD

We investigated the global registry study, ENROLL-HD, in order to analyze the largest cohort of premanifest HD patients, family members of affected HD patients, as well as genotype-negative participants. Enroll-HD is a global clinical research platform designed to facilitate clinical research into HD. Core datasets are collected annually from all research participants as part of this global multi-center longitudinal observational study. Data are monitored for quality and accuracy using a risk-based monitoring approach. All sites are required to obtain and maintain local ethical approval. We investigated the periodic dataset five (PDS5), as described previously [33,34] Ethics approval was obtained by the local ethics committee of Ruhr-University Bochum (No. 4941-14). Participants were categorized at enrollment of study entry (baseline visit) as pre-manifest/pre-motor-manifest HD, manifest/motor-manifest HD (with a diagnostic confidence level of 4 having unequivocal signs of clinical manifest HD), or as family controls. Community controls were not included in the study. Participants without an earlier conducted molecular-genetic testing prior to study participation but with first-degree relatives of an HD patient were initially included as “genetic unknown” and automatically mapped to the pre-manifest or family control-group in the dataset after genetic testing as part of the baseline visit. 

### 2.2. Measures and Statistical Analyses 

Fundamental demographic and genetic parameters were assessed in groups analyzing cytosine-adenine-guanine (CAG)-repeat lengths in the Huntingtin-gene (*HTT*), age, sex, educational level, and motoric parameters were assessed using the UHDRS—total motor score. Cognitive performance was evaluated and compared between groups with the ENROLL-HD test battery, including five cognitive tests: symbol digit modality test (SDMT), verbal fluency test (category; Verfct), stroop color naming (SCN), stroop-word reading (SWR), stroop interference test (SIT) and mini mental state examination (MMSE). Functionality was analyzed with the UHDRS-total functional capacity (TFC) and independence scale (IS). We additionally analyzed psychiatric behavior using assessments within the Problem Behaviours Assessment-short (PBA-s) questionnaire, as reported by the clinical rater, including subscores for depression, irritability/aggression, psychosis, apathy, executive function, and self-reported assessments using the Hospital Anxiety and Depression Scale/Snaith Irritability Scale (HADS-SIS)—a combined questionnaire with subscores for anxiety, depression, irritability, and outward and inward irritability.

Data were analyzed by comparing means and standard deviations in and between groups using ANCOVA analyses, controlling for age and education as a cross-sectional approach. Baseline data of study participation were analyzed in IBM SPSS Statistics V.28 (Armonk, NY, USA). Post hoc Tukey HSD testing was performed for analyzing pair-wise differences between groups. Descriptive data were depicted by chi-square testing.

## 3. Results

### 3.1. Cohorts of Pre-Manifest, Family Controls and Genotype-Negative Participants in the ENROLL-HD Database

**Figure 1:**
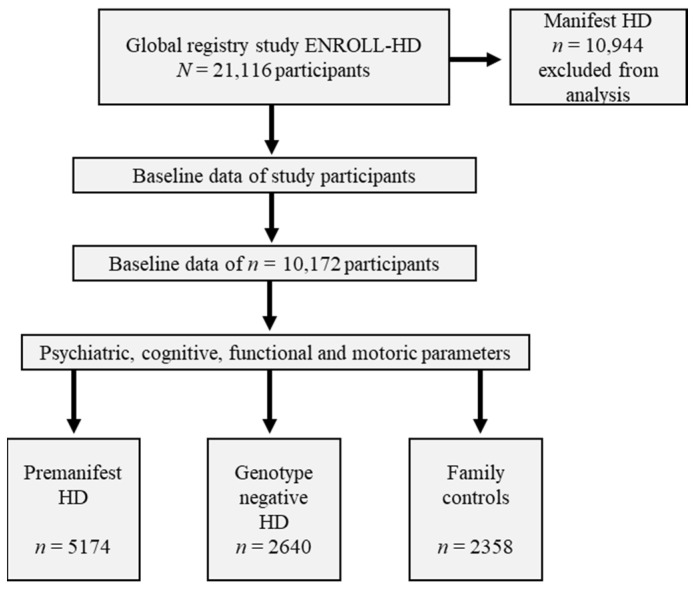
Workflow for assessing premanifest, genotype negative and family controls within the real-world dataset. Abbreviations: *n*/*N*: number; PDS-5: HD: Huntington’s disease.

Out of *N* = 21,116 participants within the global ENROLL-HD registry study, we identified a cohort of *n* = 5174 participants categorized with a premanifest motor-phenotype, *n* = 2640 with negative genotype, and *n* = 2358 as family controls (Figure 1). 

Community controls were not included in the dataset and participants introducing an unknown genotype were automatically mapped to premanifest or family control groups after the initial pseudonymized genetic testing.

### 3.2. Baseline Characteristics of Premanifest, Genotype Negative and Family Controls

As a first approach, we depicted sociodemographic data and baseline performance of motoric, cognitive, and psychiatric manifestation as mean (SD) within and between the three groups. 

Analysis of variance revealed that sociodemographic and motoric data significantly differed between groups, revealing premanifest HD participants as having higher motoric UHDRS total motor scores (all *p* < 0.001). Regarding further analyses of functional, cognitive, and psychiatric performance, we controlled for age and education to minimize the effects of underlying heterogeneous sociodemographic data. Within the large cohort of *N* = 10,172 participants, premanifest HD patients had medium lower functional scores, revealing more impairment (TFC; IS; all *p* < 0.001) of performance (Table 1). 

Regarding cognitive assessments, *n* = 2640 genotype negative participants reached the highest scores in mean, indicating better performances and significant group differences between the depicted cohorts (all *p* < 0.001). 

With regard to psychiatric parameters, the PBA-s questionnaire—as investigated by a clinical rater—revealed that premanifest HD patients suffered from higher psychiatric subscores for depression, irritability/aggression, psychosis, apathy, and executive function in the medium subscore (all *p* < 0.001). The main symptoms with highest medium subscores were identified in all three subgroups for depression and irritability.

Self-reported psychiatric assessments within the HADS-SIS-testing showed the highest scores in the medium subscore regarding anxiety, irritability, and outward and inward irritability within the premanifest HD patients and there were significant differences between groups (all *p* < 0.001). The combined questionnaire revealed the highest subscore for depression in *n =* 1755 (participants as family controls) and likewise there were significant differences between the groups (*p* < 0.001).

### 3.3. Pairwise Post Hoc Analysis between Groups

Having established analyses of cross-sectional mean data, we performed a pairwise group analysis with regard to motoric, functional, cognitive, and psychiatric performance. 

The motoric and functional analyses proved that group differences depicted within the baseline data differed based on premanifest participants with significantly higher motoric scores and less functional capacities if compared to family control and genotype-negative participants (all *p* < 0.001). No significant differences were observed for family controls compared to genotype-negative participants regarding motoric and functional performance. 

Similar differences were observed in cognitive tests showing premanifest HD with less capacities if compared to genotype-negative patients (all *p* < 0.001). Except for the SDMT, significant group differences were observed in terms of cognitive capacities in premanifest patients vs. family controls. Additionally, the large cohort of *n* = 2640 genotype negative participants showed higher scores with better cognitive performances if compared to the *n* = 2358 family controls (all *p* < 0.001) while controlling for age and education as co-variables.

Psychiatric parameters in PBA-s revealed higher group domain scores for depression, irritability/aggression, apathy, and executive function in the premanifest group, compared to genotype-negative and family members (all *p* < 0.001). The judgement of the clinical rater within the PBA-s did not depict differences regarding depression and irritability in family controls vs. genotype negative participants, but minor scores for psychosis, apathy, and executive function in family controls (*p* < 0.001).

Self-reported assessments depicted—in the Hospital Anxiety and Depression Scale/Snaith Irritability Scale—revealed more psychiatric impairments in premanifest participants if compared to genotype negative and family members, except for the depression subscore in which family members reported significantly more impairment if compared to both other groups (Table 2).

### 3.4. Exploration of Psychiatric Symptoms in Premanifest and Genotype Negative Participants in a Lifetime

As an additional approach, we analyzed, within subgroups of premanifest and genotype negative participants, how many suffered from psychiatric manifestations in their lifetime. 

During baseline assessment, investigators reported if depression (including treatment with antidepressants, with or without a formally stated diagnosis of depression), irritability, violent or aggressive behavior, apathy, perseverative obsessive behaviors, psychosis (hallucinations or delusions), have ever been part of the participant’s medical history. 

Assessments were integrated within the ENROLL-HD database solely for premanifest and genotype-negative participants revealing depression (54.1%) and irritability (40.1%) as most common psychiatric symptoms in premanifest respectively genotype-negative (depression 38.5%; irritability 34.9%) participants (Table 3).

## 4. Discussion

To investigate the disease burden in caregivers and premanifest HD patients, we took advantage of the largest database of HD patients and analyzed the global ENROLL-HD registry study, using *N* = 21,116 participants from periodic dataset five. Manifold research approaches have been attempted within the last few years, focusing on the course of the disease in manifest HD patients or investigated prevalence data, biomarkers, and outcome measures in motor-manifest and premanifest HD within the clinical research platform [6,35,36,37,38,39]. With our research, we changed the focus to a large cohort of more than *n* = 2300 participants included as family controls—coming from the direct family environment of an affected HD patient—and a further *n* = 2640 participants implemented as genotype-negative HD as a control group. In addition, we analyzed more than five thousand participants implemented as premanifest HD, potentially also affected by one parent suffering from HD. Focusing on the large cohort of premanifest participants, we investigated in these patients, formally without a clinical diagnosis of HD, more motoric, functional, cognitive, and psychiatric impairments if compared to genotype negative, and, in some parts, if compared to family controls. These findings go along with other research describing prodromal phases of HD, before the onset of distinct motoric symptoms (defined as diagnostic confidence level 4) and also go along with earlier observed neurocognitive, neuropsychiatric, biomarker, and structural MRI changes with brain atrophy in premanifest HD patients [40,41,42,43,44,45] Recently, CSF neurofilament light protein (NfL) was identified as a potential biomarker and is elevated far before the onset in pre-HD as a sensitive measure for neurodegenerative processes, starting decades before clinical onset [46]. As a limitation, no CSF measures or neuro-imaging data were available within the investigated cohorts, so neurobiological correlates could not be identified. However, further aspects of, especially, neuropsychiatric data were analyzed with a strong focus on clinical data. Premanifest HD patients suffered significantly more from depression, irritability, psychosis, and apathy, and had fewer executive functions, assessed by the clinical rater within the PBA-s scale. Additional findings were confirmed for the HADS-SIS scale. Remarkably, we observed significantly higher self-reported depression by family controls if compared to genotype-negative and premanifest HD patients. One might hypothesize that these findings reflect an enormous caregiver burden of people caring for affected HD patients, which has been reported earlier in smaller cohorts [10,12,13]. Discrepancies in HADS-SIS as a self-reported measure and PBA-s as an assessment of the clinical rater within the depression subscale might be explainable because: (i) of the different self-awareness of deficits in HD [47,48] even in prodromal HD [6]; (ii) based on an expected divergent rating of the clinical rater for premanifest HD patients; or (iii) based on family members not reporting their distress. One might hypothesize that caregivers—participating in the study as family controls—do not report their own problems during an ENROLL-HD visit to not burden their sick partner with additional worries. Hence, it might be speculative that there is a primary focus within the clinical rating on manifest or premanifest HD patients providing higher scorings in affected patients rather than in accompanying family controls or other effects of clinometric properties regarding clinical assessments had an influence, as reported earlier [49]. Such differences between clinician-rated and patient-reported outcomes were identified in earlier research [50,51]. Here, an advantage of our investigation is that both self-reported and clinician-reported assessments of psychiatric deficits were assessed. As another advantage, data regarding symptoms—not only currently reported during a baseline visit—but within the whole lifetime of the premanifest and genotype-negative participants were analyzed. As an explorative approach, we identified that 54.1% of premanifest HD patients suffered from depression, 40.1% reported irritability, 28% apathy, more than 25% persevering obsessive behaviors, 21.1% irritability and 3.5% had psychosis symptoms in their medical history. These findings are very similar to earlier meta-research about psychopathologic symptoms describing frequent symptoms with depression, anxiety, irritability or apathy (33–76%), obsessive–compulsive symptoms (10–52%), and psychotic manifestations (3–11%) in HD gene carriers [52]. These are huge impacts on psychological difficulties, on the one hand, and there is limited knowledge about beneficial individual intervention strategies, on the other, which reflect an urgent need for further research concerning useful targeted psychological therapeutic approaches [14].

Because psychopathological data are very congruent with earlier findings, we feel confident that other evaluated assessments of cognitive, psychiatric, and functional data will result in realistic findings. Conspicuously, 38.5% of genotype-negative participants suffered from depression and more than 34% suffered from irritability in their lifetimes as the two most frequent psychiatric symptoms, which seems highly frequent. These findings are congruent with depression as the most common symptom, analyzed in validations of the HADS, within normal-population cohorts [53,54], very common late- life depression [55], and especially the impact of a positive HD family history in investigated genotype negative controls within our study [56]. To investigate these effects in more detail, further information about the genotype-negative cohort would have been useful, e.g., how many in the cohort were aware of the negative genotype and how many were still “at risk”, without knowing their own molecular–genetic results. However, this information is not available in the periodic ENROLL-HD dataset. 

As a further limitation, no information about psychiatric symptoms regarding lifespan are given within the database for family controls, which would have been helpful to analyze caregiver burden in more detail. In this context, further information about specific family structures, disease stage of affected patients, or specific symptoms, in particular relating to greater or less caregiver burden, could have been helpful within the dataset. For data-protection reasons, it is currently not possible to link specific symptoms in an affected parent, e.g., aggressive behavior, to, e.g., a genotype-negative child by their complaints. As an advantage—regarding the clinical focus of the database—we additionally analyzed the cognitive data of described cohorts. In particular, we investigated significantly less cognitive capacities in all cognitive tests in family controls when compared to genotype-negative participants. We statistically controlled for diverse fundamental sociodemographic parameters of age and education to minimize a potential bias for older family controls, since age is known to have a negative impact on cognitive capacities [57]. Remarkably, after controlling for covariates, the effects remained. One might thereafter hypothesize—as a potential explanation—that being a family control for an affected HD patient goes along with cognitive impairments, possibly due to a more loaded or stressful situation, seen in other investigations with smaller cohorts [10,13,15] As a further explanation, stressful situations for caregivers along with burdens and depressive symptoms, might have led to cognitive disorders, which has been described earlier as pseudodementia [58]. However, we cannot entirely exclude loaded family situations or even disease burden in gene-negative participants coming from an HD family. To validate these findings, further investigations with large cohorts of family controls with a more detailed focus, using further questionnaires or investigating specific relationship components, are necessary.

## 5. Conclusions

In summary, we analyzed the largest cohort of HD patients and families, and we can confirm previous research describing slight impairments in motoric, functional, cognitive, and psychiatric components in premanifest HD as a prodromal stadium of early changes due to HD pathology. As a second approach, we investigated HD family controls suffering from higher self-reported depression when compared to premanifest HD and less cognitive capacities when compared to genotype-negative controls within the study. As an explanation, cognitive impairment was potentially caused by loaded or stressful situations in HD families and especially in relatives who care for a manifest HD patient. With our research, we aim to sensitize ENROLL-HD investigators so that they are aware of caregiver burdens caused by HD and encourage support with socio-medical care. In particular, further surveys and variables can be helpful within the database to identify caregivers at risk and provide adequate psychological interventions.

## Figures and Tables

**Table 1:** Baseline sociodemographic, motoric, functional, cognitive and psychiatric data between groups at baseline visit. Cross-sectional data revealed group differences between premanifest, genotype-negative and family control participants within the ENROLL-HD study. +: higher scores = better performance; #: higher scores = more impairment.

	Premanifest HD (*n* = 5174)	Family Controls (*n* = 2358)	Genotype Negative (*n* = 2640)	F	*p*	Part. Eta2
Age (y); M (SD)	39.8 (12.1)	52.7 (12.9)	41.3 (14.2)	858.832	<0.001	0.145
Sex (f/m) (%f)	3099/2074 (59.9)	1339/1018 (56.8)	1717/922 (65.1)	37.395	<0.001	0.001
CAG high	42.4 (2.8)	20.0 (3.4)	20.3 (3.8)	60,637.636	<0.001	0.923
ISCED+	3.9 (1.1) (*n* = 5157)	3.8 (1.2) (*n* = 2351)	3.9 (1.2) (*n* = 2624)	14.737	<0.001	0.003
Motoric UHDRS TMS #	3.0 (4.5)	1.5 (2.9)	1.7 (3.6)	167.799	<0.001	0.031
TFC+	12.7 (0.9)	12.9 (0.6)	12.9 (0.7)	43.321	<0.001	0.008
IS+	98.9 (3.8)	99.6 (2.1)	99.6 (2.8)	43.639	<0.001	0.009
SDMT+	49.3 (12.1) (*n* = 5139)	47.8 (12.0) (*n* = 2342)	51.7 (11.9) (*n* = 2610)	105.016	<0.001	0.020
Verfct+	21.2 (5.8) (*n* = 5133)	21.7 (5.5) (*n* = 2335)	22.1 (5.7) (*n* = 2618)	58.897	<0.001	0.012
SCNT+	72.4 (14.8) (*n* = 5126)	73.1 (14.1) (*n* = 2324)	75.4 (14.3) (*n* = 2606)	91.273	<0.001	0.018
SWRT+	92.7 (18.4) (*n* = 5130)	94.3 (17.3) (*n* = 2326)	96.6 (17.3) (*n* = 2611)	92.996	<0.001	0.018
MMSE+	28.7 (1.7) (*n* = 3653)	28.8 (1.6) (*n* = 1612)	29.0 (1.5) (*n* = 2031)	56.640	<0.001	0.015
PBA_depscore #	4.3 (5.8) (*n* = 5155)	3.5 (4.9) (*n* = 2352)	3.2 (5.2) (*n* = 2631)	39.697	<0.001	0.008
Irascore #	2.0 (3.6)	1.4 (2.7)	1.2 (2.8)	54.574	<0.001	0.011
Psyscore #	0.12 (1.0)	0.02 (0.3)	0.11 (0.9)	5.531	0.004	0.001
Aptscore #	0.99 (2.4)	0.43 (1.4)	0.51 (1.7)	71.966	<0.001	0.014
Exfscore #	1.3 (3.1)	0.6 (2.1)	0.9 (2.4)	37.343	<0.001	0.007
Hads_anxscore #	5.6 (4.0) (*n* = 3552)	5.3 (3.7) (*n* = 1755)	5.2 (3.9) (*n* = 1988)	6.669	<0.001	0.002
Depscore #	3.6 (3.5)	3.9 (3.3)	3.1 (3.1)	39.697	<0.001	0.008
Irrascore #	5.1 (4.0)	4.3 (3.1)	4.3 (3.7)	32.927	<0.001	0.009
Outscore #	3.2 (2.5)	2.8 (2.1)	2.7 (2.3)	28.648	<0.001	0.008
Inwscore #	1.9 (2.1)	1.5 (1.7)	1.6 (2.0)	20.130	<0.001	0.005

Abbreviations: M: mean; SD: standard deviation; *p*: *p* value; F: F value; Part Eta^2^: effect size; y: years; UHDRS: Unified Huntington’s Disease Rating Scale; CAG: cytosine-adenine-guanine repeat length; ISCED: educational level; TMS: total motor score; TFC: total functional capacity; IS: independence scale; SDMT: symbol digit modality test; Verfct: verbal fluency test (category); SCNT: stroop color naming test; SWRT: stroop word reading test; SIT: stroop interference test; MMSE: mini mental state examination; PBA_depscore: Problem Behaviours Assessment-Short Depression; Irascore: irritability/aggression; Psyscore: psychosis; Aptscore: apathy; Exfscore: executive function; Hads_anxscore: Hospital Anxiety and Depression Scale_anxiety subscore; depscore: depression subscore; Irrscore: irritability subscore; outscore: outward irritability subscore; Inwscore: inward irritability subscore. Values highlighted (background colors): green = best performance; red = most impairment.

**Table 2:** Post hoc Tukey-HSD testing for motoric, functional and psychiatric parameters assessing pairwise differences between groups. +: higher scores = better performance; #: higher scores = more impairment.

Post-Hoc Tukey-HSD	Participant Category 1	vs. Category 2	Sign. (*p*)
Motoric UHDRS TMS#	Premanifest HD	Genotype negative	<0.001
		Family controls	<0.001
	Genotype negative	Family controls	0.325
ISCED+	Premanifest HD	Genotype negative	<0.001
		Family controls	<0.001
	Genotype negative	Family controls	0.325
TFC+	Premanifest HD	Genotype negative	<0.001
		Family controls	<0.001
	Genotype negative	Family controls	0.520
IS+	Premanifest HD	Genotype negative	0.333
		Family controls	<0.001
	Genotype negative	Family controls	0.060
SDMT+	Premanifest HD	Genotype negative	<0.001
		Family controls	<0.001
	Genotype negative	Family controls	<0.001
Verfct+	Premanifest HD	Genotype negative	<0.001
		Family controls	<0.005
	Genotype negative	Family controls	<0.050
SCNT+	Premanifest HD	Genotype negative	<0.001
		Family controls	0.096
	Genotype negative	Family controls	<0.001
SWRT+	Premanifest HD	Genotype negative	<0.001
		Family controls	<0.005
	Genotype negative	Family controls	<0.001
MMSE+	Premanifest HD	Genotype negative	<0.001
		Family controls	<0.05
	Genotype negative	Family controls	<0.001
PBA_depscore #	Premanifest HD	Genotype negative	<0.001
		Family controls	<0.001
	Genotype negative	Family controls	0.233
Irascore#	Premanifest HD	Genotype negative	<0.001
		Family controls	<0.001
	Genotype negative	Family controls	0.167
Psyscore#	Premanifest HD	Genotype negative	0.756
		Family controls	<0.001
	Genotype negative	Family controls	<0.005
Aptscore#	Premanifest HD	Genotype negative	<0.001
		Family controls	<0.001
	Genotype negative	Family controls	<0.001
Exfscore#	Premanifest HD	Genotype negative	<0.001
		Family controls	<0.001
	Genotype negative	Family controls	<0.001
Hads_anxscore#	Premanifest HD	Genotype negative	<0.001
		Family controls	<0.05
	Genotype negative	Family controls	0.842
Depscore#	Premanifest HD	Genotype negative	<0.001
		Family controls	<0.05
	Genotype negative	Family controls	<0.001
Irrascore#	Premanifest HD	Genotype negative	<0.001
		Family controls	<0.001
	Genotype negative	Family controls	0.960
Outscore#	Premanifest HD	Genotype negative	<0.001
		Family controls	<0.001
	Genotype negative	Family controls	0.874
Inwscore#	Premanifest HD	Genotype negative	<0.001
		Family controls	<0.001
	Genotype negative	Family controls	0.534

Abbreviations: M: mean; SD: standard deviation; *p*: *p* value; y: years; UHDRS: Unified Huntington’s Disease Rating Scale; TMS: total motor score; ISCED: educational level; TFC: total functional capacity; IS: independence scale; SDMT: symbol digit modality test; Verfct: verbal fluency test (category); SCNT: stroop color naming test; SWRT: stroop word reading test; SIT: stroop interference test; MMSE: mini mental state examination; PBA_depscore: Problem Behaviours Assessment- Short Depression; Irascore: irritability/aggression; Psyscore: psychosis; Aptscore: apathy; Exfscore: executive function; Hads_anxscore: Hospital Anxiety and Depression Scale_anxiety subscore; depscore: depression subscore; Irrscore: irritability subscore; outscore: outward irritability subscore; Inwscore: inward irritability subscore.

**Table 3:** Explorative description of psychiatric symptoms being part of participants medical history in a lifetime.

	Premanifest HD	Genotype Negative
	(*n* = 5174)	(*n* = 2640)
Depression		
Yes (%)	2797 (54.1)	1016 (38.5)
No (%)	2375 (45.9)	1622 (61.5)
Irritability		
Yes (%)	2074 (40.1)	922 (34.9)
No (%)	3099 (59.9)	1717 (65.1)
Aggressive behavior		
Yes (%)	1091 (21.1)	262 (9.9)
No (%)	4081 (78.9)	2376 (90.1)
Apathy		
Yes (%)	1447 (28.0)	358 (13.6)
No (%)	3724 (72.0)	2280 (86.4)
Obsessive behavior		
Yes (%)	1302 (25.2)	354 (13.4)
No (%)	3869 (74.8)	2283 (86.6)
Psychosis		
Yes (%)	181 (3.5)	61 (2.3)
No (%)	4990 (96.5)	2576 (97.7)

## Data Availability

The data that support the findings of this study are available from the corresponding author upon reasonable request.

## References

[1] Walker F.O. (2007). Huntington’s disease. Lancet.

[2] Ghosh R., Tabrizi S.J. (2018). Huntington disease. Handb. Clin. Neurol..

[3] Achenbach J., von Hein S.M., Saft C. (2020). Functional and cognitive capacity differ in dystonic motor subtypes when compared to choreatic and hypokinetic-rigid motor subtypes in Huntington’s disease. Brain Behav..

[4] Saft C., Lauter T., Kraus P.H., Przuntek H., Andrich J.E. (2006). Dose-dependent improvement of myoclonic hyperkinesia due to Valproic acid in eight Huntington’s Disease patients: A case series. BMC Neurol..

[5] Roos R.A.C. (2010). Huntington’s disease: A clinical review. Orphanet J. Rare Dis..

[6] Epping E.A., Kim J.-I., Craufurd D., Brashers-Krug T.M., Anderson K.E., McCusker E., Luther J., Long J.D., Paulsen J.S. (2016). Longitudinal Psychiatric Symptoms in Prodromal Huntington’s Disease: A Decade of Data. Am. J. Psychiatry.

[7] Mestre T.A., Shannon K. (2017). Huntington disease care: From the past to the present, to the future. Parkinsonism Relat. Disord..

[8] Banaszkiewicz K., Sitek E.J., Rudzińska M., Sołtan W., Sławek J., Szczudlik A. (2012). Huntington’s disease from the patient, caregiver and physician’s perspectives: Three sides of the same coin?. J. Neural Transm..

[9] Røthing M., Malterud K., Frich J.C. (2014). Caregiver roles in families affected by Huntington’s disease: A qualitative interview study. Scand. J. Caring Sci..

[10] Hergert D.C., Cimino C.R. (2021). Predictors of Caregiver Burden in Huntington’s Disease. Arch. Clin. Neuropsychol..

[11] Jona C.M.H., Labuschagne I., Mercieca E.-C., Fisher F., Gluyas C., Stout J.C., Andrews S.C. (2017). Families Affected by Huntington’s Disease Report Difficulties in Communication, Emotional Involvement, and Problem Solving. J. Huntingtons. Dis..

[12] Sellers J., Ridner S.H., Claassen D.O. (2020). A Systematic Review of Neuropsychiatric Symptoms and Functional Capacity in Huntington’s Disease. J. Neuropsychiatry Clin. Neurosci..

[13] Yu M., Tan K., Koloms K., Bega D. (2019). Assessment of Caregiver Burden in Huntington’s Disease. J. Huntingtons. Dis..

[14] Zarotti N., Dale M., Eccles F., Simpson J. (2020). Psychological Interventions for People with Huntington’s Disease: A Call to Arms. J. Huntingtons Dis..

[15] Wibawa P., Zombor R., Dragovic M., Hayhow B., Lee J., Panegyres P.K., Rock D., Starkstein S.E. (2020). Anosognosia Is Associated with Greater Caregiver Burden and Poorer Executive Function in Huntington Disease. J. Geriatr. Psychiatry Neurol..

[16] Thompson J.C., Harris J., Sollom A.C., Stopford C.L., Howard E., Snowden J.S., Craufurd D. (2012). Longitudinal evaluation of neuropsychiatric symptoms in Huntington’s disease. J. Neuropsychiatry Clin. Neurosci..

[17] Thompson J.C., Snowden J.S., Craufurd D., Neary D. (2002). Behavior in Huntington’s disease: Dissociating cognition-based and mood-based changes. J. Neuropsychiatry Clin. Neurosci..

[18] Migliore S., D’Aurizio G., Maffi S., Ceccarelli C., Ristori G., Romano S., Castaldo A., Mariotti C., Curcio G., Squitieri F. (2021). Cognitive and behavioral associated changes in manifest Huntington disease: A retrospective cross-sectional study. Brain Behav..

[19] Simpson J., Dale M., Theed R., Gunn S., Zarotti N., Eccles F.J.R. (2019). Validity of irritability in Huntington’s disease: A scoping review. Cortex.

[20] Brüne M., Blank K., Witthaus H., Saft C. (2011). “Theory of mind” is impaired in Huntington’s disease. Mov. Disord..

[21] Saft C., Lissek S., Hoffmann R., Nicolas V., Tegenthoff M., Juckel G., Brüne M. (2013). Mentalizing in preclinical Huntington’s disease: An fMRI study using cartoon picture stories. Brain Imaging Behav..

[22] Brüne M., von Hein S.M., Claassen C., Hoffmann R., Saft C. (2021). Altered third-party punishment in Huntington’s disease: A study using neuroeconomic games. Brain Behav..

[23] Johnson S.A., Stout J.C., Solomon A.C., Langbehn D.R., Aylward E.H., Cruce C.B., Ross C.A., Nance M., Kayson E., Julian-Baros E. (2007). Beyond disgust: Impaired recognition of negative emotions prior to diagnosis in Huntington’s disease. Brain.

[24] Cavallo M., Sergi A., Pagani M. (2021). Cognitive and social cognition deficits in Huntington’s disease differ between the prodromal and the manifest stages of the condition: A scoping review of recent evidence. Br. J. Clin. Psychol..

[25] Martinez-Horta S., Perez-Perez J., van Duijn E., Fernandez-Bobadilla R., Carceller M., Pagonabarraga J., Pascual-Sedano B., Campolongo A., Ruiz-Idiago J., Sampedro F. (2016). Neuropsychiatric symptoms are very common in premanifest and early stage Huntington’s Disease. Parkinsonism Relat. Disord..

[26] Vaccarino A.L., Sills T., Anderson K.E., Bachoud-Lévi A.-C., Borowsky B., Craufurd D., Duff K., Giuliano J., Groves M., Guttman M. (2011). Assessment of depression, anxiety and apathy in prodromal and early huntington disease. PLoS Curr..

[27] Butėnaitė A., Strumila R., Lengvenytė A., Pakutkaitė I.K., Morkūnienė A., Matulevičienė A., Dlugauskas E., Utkus A. (2021). Significant Association Between Huntingtin Gene Mutation and Prevalence of Hopelessness, Depression and Anxiety Symptoms. Acta Med. Litu..

[28] Van Duijn E., Giltay E.J., Zitman F.G., Roos R.A.C., van der Mast R.C. (2010). Measurement of psychopathology in Huntington’s disease: The critical role of caregivers. J. Nerv. Ment. Dis..

[29] Kingma E.M., van Duijn E., Timman R., van der Mast R.C., Roos R.A.C. (2008). Behavioural problems in Huntington’s disease using the Problem Behaviours Assessment. Gen. Hosp. Psychiatry.

[30] Dale M., Maltby J., Martucci R., Shimozaki S. (2015). Factor analysis of the hospital anxiety and depression scale among a Huntington’s disease population. Mov. Disord..

[31] Callaghan J., Stopford C., Arran N., Boisse M.-F., Coleman A., Santos R.D., Dumas E.M., Hart E.P., Justo D., Owen G. (2015). Reliability and factor structure of the Short Problem Behaviors Assessment for Huntington’s disease (PBA-s) in the TRACK-HD and REGISTRY studies. J. Neuropsychiatry Clin. Neurosci..

[32] McNally G., Rickards H., Horton M., Craufurd D. (2015). Exploring the Validity of the Short Version of the Problem Behaviours Assessment (PBA-s) for Huntington’s disease: A Rasch Analysis. J. Huntingtons. Dis..

[33] Achenbach J., Faissner S., Saft C. (2021). Differential Diagnosis of Chorea-HIV Infection Delays Diagnosis of Huntington’s Disease by Years. Brain Sci..

[34] Achenbach J., Saft C., Faissner S. (2021). Longitudinal Evaluation of the Effect of Tricyclic Antidepressants and Neuroleptics on the Course of Huntington’s Disease—Data from a Real World Cohort. Brain Sci..

[35] Sathe S., Ware J., Levey J., Neacy E., Blumenstein R., Noble S., Mühlbäck A., Rosser A., Landwehrmeyer G.B., Sampaio C. (2021). Enroll-HD: An Integrated Clinical Research Platform and Worldwide Observational Study for Huntington’s Disease. Front. Neurol..

[36] Achenbach J., Saft C. (2021). Data from ENROLL-HD: Is the prevalence of juvenile and pediatric Huntington’s disease overestimated?. Parkinsonism Relat. Disord..

[37] Crowell V., Houghton R., Tomar A., Fernandes T., Squitieri F. (2021). Modeling Manifest Huntington’s Disease Prevalence Using Diagnosed Incidence and Survival Time. Neuroepidemiology.

[38] Sprenger G.P., Roos R.A.C., van Zwet E., Reijntjes R.H., Achterberg W.P., de Bot S.T. (2021). The prevalence of pain in Huntington’s disease in a large worldwide cohort. Parkinsonism Relat. Disord..

[39] Tabrizi S.J., Scahill R.I., Owen G., Durr A., Leavitt B.R., Roos R.A., Borowsky B., Landwehrmeyer B., Frost C., Johnson H. (2013). Predictors of phenotypic progression and disease onset in premanifest and early-stage Huntington’s disease in the TRACK-HD study: Analysis of 36-month observational data. Lancet Neurol..

[40] Wijeratne P.A., Garbarino S., Gregory S., Johnson E.B., Scahill R.I., Paulsen J.S., Tabrizi S.J., Lorenzi M., Alexander D.C. (2021). Revealing the Timeline of Structural MRI Changes in Premanifest to Manifest Huntington Disease. Neurol. Genet..

[41] Winder J.Y., Roos R.A.C. (2018). Premanifest Huntington’s disease: Examination of oculomotor abnormalities in clinical practice. PLoS ONE.

[42] Scahill R.I., Andre R., Tabrizi S.J., Aylward E.H. (2017). Structural imaging in premanifest and manifest Huntington disease. Handb. Clin. Neurol..

[43] Heim B., Peball M., Saft C., von Hein S.M., Ellmerer P., Piater J.M., Seppi K., Djamshidian A. (2020). Time will tell: Decision making in premanifest and manifest Huntington’s disease. Brain Behav..

[44] You S.C., Geschwind M.D., Sha S.J., Apple A., Satris G., Wood K.A., Johnson E.T., Gooblar J., Feuerstein J.S., Finkbeiner S. (2014). Executive functions in premanifest Huntington’s disease. Mov. Disord..

[45] Paulsen J.S., Miller A.C., Hayes T., Shaw E. (2017). Cognitive and behavioral changes in Huntington disease before diagnosis. Handb. Clin. Neurol..

[46] Scahill R.I., Zeun P., Osborne-Crowley K., Johnson E.B., Gregory S., Parker C., Lowe J., Nair A., O’Callaghan C., Langley C. (2020). Biological and clinical characteristics of gene carriers far from predicted onset in the Huntington’s disease Young Adult Study (HD-YAS): A cross-sectional analysis. Lancet Neurol..

[47] Júlio F., Ribeiro M.J., Morgadinho A., Sousa M., van Asselen M., Simões M.R., Castelo-Branco M., Januário C. (2020). Cognition, function and awareness of disease impact in early Parkinson’s and Huntington’s disease. Disabil. Rehabil..

[48] Hergert D.C., Sanchez-Ramos J., Cimino C.R. (2020). Awareness of Chorea in Huntington’s Disease. J. Huntingtons. Dis..

[49] Abreu D., Ware J., Georgiou-Karistianis N., Leavitt B.R., Fitzer-Attas C.J., Lobo R., Fernandes A.R., Handley O., Anderson K.E., Stout J.C. (2021). Utility of Huntington’s Disease Assessments by Disease Stage: Floor/Ceiling Effects. Front. Neurol..

[50] Carlozzi N.E., Boileau N.R., Perlmutter J.S., Chou K.L., Stout J.C., Paulsen J.S., McCormack M.K., Cella D., Nance M.A., Lai J.-S. (2018). Agreement between clinician-rated versus patient-reported outcomes in Huntington disease. J. Neurol..

[51] Winder J.Y., Roos R.A.C., Burgunder J.-M., Marinus J., Reilmann R. (2018). Interrater Reliability of the Unified Huntington’s Disease Rating Scale-Total Motor Score Certification. Mov. Disord. Clin. Pract..

[52] Van Duijn E., Kingma E.M., van der Mast R.C. (2007). Psychopathology in verified Huntington’s disease gene carriers. J. Neuropsychiatry Clin. Neurosci..

[53] Hinz A., Schwarz R. (2001). Angst und Depression in der Allgemeinbevölkerung. Eine Normierungsstudie zur Hospital Anxiety and Depression Scale. Psychother. Psychosom. Med. Psychol..

[54] Herrmann C. (1997). International experiences with the Hospital Anxiety and Depression Scale—A review of validation data and clinical results. J. Psychosom. Res..

[55] Luppa M., Sikorski C., Luck T., Ehreke L., Konnopka A., Wiese B., Weyerer S., König H.-H., Riedel-Heller S.G. (2012). Age- and gender-specific prevalence of depression in latest-life—Systematic review and meta-analysis. J. Affect. Disord..

[56] Kringlen G., Kinsley L., Aufox S., Rouleau G., Bega D. (2017). The Impact of Family History on the Clinical Features of Huntington’s Disease. J. Huntingtons. Dis..

[57] Crum R.M., Anthony J.C., Bassett S.S., Folstein M.F. (1993). Population-based norms for the Mini-Mental State Examination by age and educational level. JAMA.

[58] Sekhon S., Marwaha R. (2021). StatPearls: Depressive Cognitive Disorders.

